# Mesoporous Properties of Bioactive Glass Synthesized by Spray Pyrolysis with Various Polyethylene Glycol and Acid Additions

**DOI:** 10.3390/polym13040618

**Published:** 2021-02-18

**Authors:** Tzu-Yu Peng, Pei-Yun Tsai, May-Show Chen, Yuichi Mine, Shan-Hua Wu, Chin-Yi Chen, Dan-Jae Lin, Chung-Kwei Lin

**Affiliations:** 1 School of Dentistry, College of Dentistry, China Medical University, Taichung 404, Taiwan; pengtzuyu1014@gmail.com (T.-Y.P.); u105000004@cmu.edu.tw (P.-Y.T.); djlin@mail.cmu.edu.tw (D.-J.L.); 2 Research Center of Digital Oral Science and Technology, College of Oral Medicine, Taipei Medical University, Taipei 110, Taiwan; maychen@tmu.edu.tw (M.-S.C.); chencyi@fcu.edu.tw (C.-Y.C.); 3 Department of Anatomy and Functional Restorations, Graduate School of Biomedical and Health Sciences, Hiroshima University, Hiroshima 734-8553, Japan; 4 School of Dentistry, College of Oral Medicine, Taipei Medical University, Taipei 110, Taiwan; 5 Division of Prosthodontics, Department of Dentistry, Taipei Medical University Hospital, Taipei 110, Taiwan; 6 Department of Medical System Engineering, Graduate School of Biomedical and Health Sciences, Hiroshima University, Hiroshima 734-8553, Japan; mine@hiroshima-u.ac.jp; 7 Department of Materials Science and Engineering, Feng Chia University, Taichung 407, Taiwan; wsh3400@gmail.com; 8 School of Dental Technology, College of Oral Medicine, Taipei Medical University, Taipei 110, Taiwan; 9 Additive Manufacturing Center for Mass Customization Production, National Taipei University of Technology, Taipei 106, Taiwan

**Keywords:** mesoporous bioactive glass, spray pyrolysis processes, polyethylene glycol, in vitro bioactivity

## Abstract

Mesoporous bioactive glass (MBG) has a high specific surface area, promoting the reaction area, thereby improving the bioactivity; thus, MBG is currently gaining popularity in the biomaterial field. Spray pyrolysis (SP) is a one-pot process that has the advantages of shorter process time and better particle bioactivity, therefore, MBG was prepared by SP process with various polyethylene glycol (PEG, molecular weight ranged from 2000–12,000) and acid (HCl and CH_3_COOH) additions. In vitro bioactivity and mesoporous properties of the so-obtained MBG were investigated. The experimental results showed that all the MBG exhibited amorphous and mesoporous structure. Increasing the molecular weight of PEG can improve the mesoporous structure and bioactivity of MBG. Whereas optimized MBG was prepared with suitable concentration of PEG and CH_3_COOH. In the present work, MBG synthesized via spray pyrolysis by adding 5 g of PEG with a molecular weight of 12,000 and 50 mL of CH_3_COOH exhibited the best in vitro bioactivity and mesoporous structure.

## 1. Introduction

Bioactive glass (BG) is commonly composed of SiO_2_, CaO, and P_2_O_5_ [1,2]. BG is a surface-active bioceramics, with non-toxicity, non-inflammatory, and osteogenic potential that can firmly bond with bone and induce hard and soft tissue regeneration [3,4,5]. BG is also safe and stable in the human body [6,7]; thereby, BG is widely used in clinical application in dentistry, orthopedics, and biomedical engineering, such as bone replacement, tooth repair, and drug-carrying materials [8,9,10,11].

The fabrication process of BG includes glass melting, sol-gel, and spray pyrolysis processes [12]. Glass melting process is the conventional process that requires high temperatures (1250–1400 °C) and the product may contain impurities and further result in low bioactivity of synthetic glass [13,14]. Sol-gel process is introduced to BG synthesis in 1991 [15] and became a popular alternative method because of the relatively lower heat treatment temperature (600–800 °C) compared to the glass melting processes, and ease of control of textural properties [16,17]. Nevertheless, as with conventional glass melting processes, sol-gel process is also a discontinuous process, so the product quality is relatively unstable, and the long processing time (3–7 days) makes it inappropriate for mass production [13]. Spray pyrolysis (SP) process is an emerging process used in BG synthesis. This process is a continuous one-pot processing [12] where the instability among each batch can be minimized. Using SP process, low-cost high purity BG can be prepared at lower processing temperatures (500–600 °C) in a few hours [13,18]. Thus, SP process is considered as a potential and developmental method of BG synthesis.

In order to expand the clinical application of BG, it will be beneficial to increase the reaction areas by creating porous structures so that osteoconduction and osteoinduction can be promoted for bone regeneration. Scholars introduced the idea of adding polymer surfactant, such as P-123 (EO_20_PO_70_EO_20_) or F-127(EO_106_PO_70_EO_106_), as a structural template to fabricate mesoporous bioactive glass (MBG) to increase the specific surface area of BG effectively [18,19,20]. MBG possessing hollow, porous structure, and high specific surface area not only exhibits excellent bioactivities but also serves as drugs or bioactive molecular carriers [11,21,22]. Nevertheless, both P-123 and F-127 have non-single composition and are copolymers of PPO-PEO [PEO, poly(ethylene oxide); PPO, poly(propylene oxide)] contains lipophilic and hydrophilic polymers, thus, it is difficult conducting a subtle examination of the surfactants’ physical properties [19,20]. Reports indicate that polyethylene glycol (PEG), a linear hydrophilic polymer, has been successfully applied as a surfactant for synthesizing MBG [23,24]. PEG is an amphiphile with the ability to reduce surface activity, and has properties such as non-toxicity, excellent biocompatibility, anti-protein adsorption properties, and high stability; hence, PEG has been approved for in vivo injection and has application in cosmetics, biotechnology, and medicine [25]. Moreover, PEG can urge the particle distribution to become uniform, so it is expected to improve the shortcomings of the uneven distribution of MBG particles produced by the SP process [26]. In the present study, MBG was synthesized by the SP process where PEG was added as a surfactant under different precursor solution conditions (molecular weights of PEG, and PEG or acid concentrations). The mesoporous properties of the so-obtained MBG particles were investigated.

## 2. Materials and Methods

### 2.1. MBG Particles Synthesis

**Figure 1:**
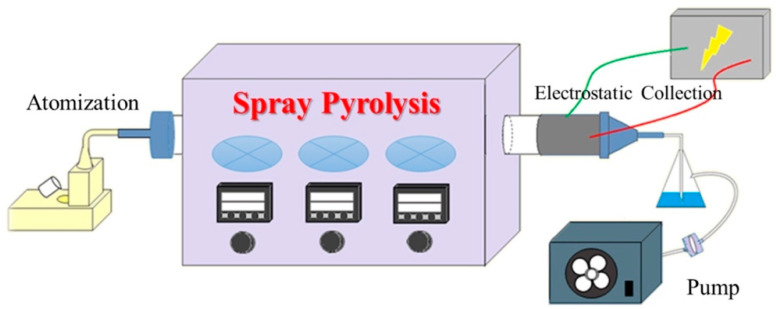
Spray pyrolysis (SP) process device diagram.

The MBG particles were synthesized through spray pyrolysis (SP) process in the present study. Figure 1 shows the schematic illustration of the SP process where the precursor solution was poured into an ultrasonic humidifier (KT-100, King Ultrasonics Co., New Taipei City, Taiwan) with a frequency of 1.65 MHz. Subsequently, the atomized droplets were brought into the furnace that was set at three different temperature zones of 200, 700, and 350 °C. After undergoing solvent evaporation, solute precipitation, and precursor decomposition, the spray pyrolyzed MBG particles were collected by an electrostatic collector with a high voltage of 16 kV. The MBG particles were obtained after 12 h quiescence and desiccation.

### 2.2. Precursor Solution Preparation Conditions

The Si-Ca-P ternary system is the most commonly used BG component in biomedical-related fields [1,2]. Silicon (Si) is essential for metabolic processes, formation, and calcification of bone tissue [27], calcium (Ca) is favored in osteoblast proliferation [28], and phosphorus (P) is a key regulator in bone formation [29]. The measured precursor materials of SiO_2_–CaO–P_2_O_5_ (the molar ratio of Si: Ca: P = 80:15:5) were prepared by dissolving 7.2 mL tetraethyl orthosilicate (TEOS), 1.4 g calcium nitrate tetrahydrate (CN), and 0.68 mL triethyl phosphate (TEP) [8,30]. The precursors were dissolved in 60 g ethanol and acid (HCl or CH_3_COOH), and then polyethylene glycol (PEG) was added as a surfactant. Deionized water was finally added and stirred to prepare a 1000 mL precursor solution. All the medicaments used in the present study are shown in Table 1. The PEG and acid addition conditions were divided into three parts in order to evaluate the in vitro bioactivity and mesoporous properties of the synthesized MBG particles. The synthesis conditions are listed in Table 2. Note that all the precursor solutions were 1000 mL and then the SP process was conducted, regardless of the synthesis conditions.

First, different molecular weights of PEG were added into the precursor solution and HCl (0.5 M) was used as the hydrolysis catalyst. The PEG addition conditions were: no addition (MEG-P0), added PEG with varying molecular weights of 2000 (MEG-P2), 4000 (MEG-P4), 8000 (MEG-P8), and 12,000 (MEG-P12).

Second, MBG particles with the best in vitro bioactivity were used from the previous part’s results (MBG-P12), and CH_3_COOH was used to replace HCl as the hydrolysis catalyst. The aim is to evaluate the influence of CH_3_COOH concentrations on mesoporous properties of MBG particles. The amounts of added CH_3_COOH were set as 25 mL (7MBG2A-P12), 50 mL (7MBG5A-P12), and 75 mL (7MBG7A-P12).

Third, CH_3_COOH concentration with the best in vitro bioactivity (7MBG5A-P12) was used from the results of the previous part, and different concentrations of PEG were added to prepare the precursor solutions. In order to evaluate the influence of PEG concentrations on mesoporous properties of MBG particles, various amounts of PEG, 3 g (3MBG5A-P12), 5 g (5MBG5A-P12), and 7 g (7MBG5A-P12) were added.

### 2.3. Characterizations of MBG Particles

The powder morphology of the MBG particles was observed by field emission scanning electron microscopy (FE-SEM; JSM-7800F, JEOL Ltd., Tokyo, Japan). The FE-SEM images were analyzed using Image J (Java 1.8.0, NIH, Bethesda, MD, USA.) to obtain the particle size distribution of the MBG particles. The MBG particles synthesized with various parameters were examined by using a multipurpose X-ray thin-film micro area diffractometer (SRAM18XHF, MacScience Co., Ltd., Tokyo, Japan) with monochromatic Cu Kα radiation (λ = 1.54 Å) operated at 40 kV, 100 mA, and scanning angles ranging from 20 to 80 degree with a scanning rate of 2 degrees 2θ/min. To prepare the particles for the morphology observation, the particles were first dispersed in ethanol in an ultrasonic bath for 20 min, and then a drop of suspension was placed onto a carbon film grid. Then the solvent on the carbon grid was evaporated under 40 W fluorescent lamp. Field emission transmission electron microscopy (FE-TEM; JEM-2010, JEOL Ltd., Tokyo, Japan) operated at 200 kV was used to characterize the internal structures and micrographs of MBG particle. The specific surface area of the MBG particles were determined by the Brunauer–Emmett–Teller (BET) method where the nitrogen adsorption and desorption isotherm data were obtained at −196 °C on a constant-volume adsorption apparatus (NovaTouch, Quantachrome Instruments, Boynton Beach, FL, USA). All the testing samples were degassed at 200 °C for 4 h prior to the tests.

### 2.4. In Vitro Bioactivity Analysis

For in vitro bioactivity analysis, the MBG particles were first subjected to acid cleaning. The procedures were as followed, first, 1 M nitric acid (HNO_3_) was added to MBG particles (MBG:HNO_3_ = 1:2) and stirred for 5 min. Then, deionized water was used for centrifugal cleaning and placed in a drying oven at 80 °C. After acid cleaning, MBG particles were immersed in the simulated body fluid (SBF) with a ratio of MBG:SBF = 1 g:50 mL. The ion concentrations of SBF were Na^+^ 142.0, K^+^ 5.0, Mg^2+^ 1.5, Ca^2+^ 2.5, Cl^−^ 147.8, HCO^3−^ 4.2, HPO_4_^2−^ 1.0, and SO_4_^2−^ 0.5 mmol/L. Further, the samples were placed in thermostatic bath at 37 °C and immersed for various times, and then centrifuged and placed it in a drying oven to dry at 80 °C. Finally, the MBG particles after SBF immersion were analyzed by XRD and FE-SEM.

## 3. Results

### 3.1. Different Molecular Weights of PEG

**Figure 2:**
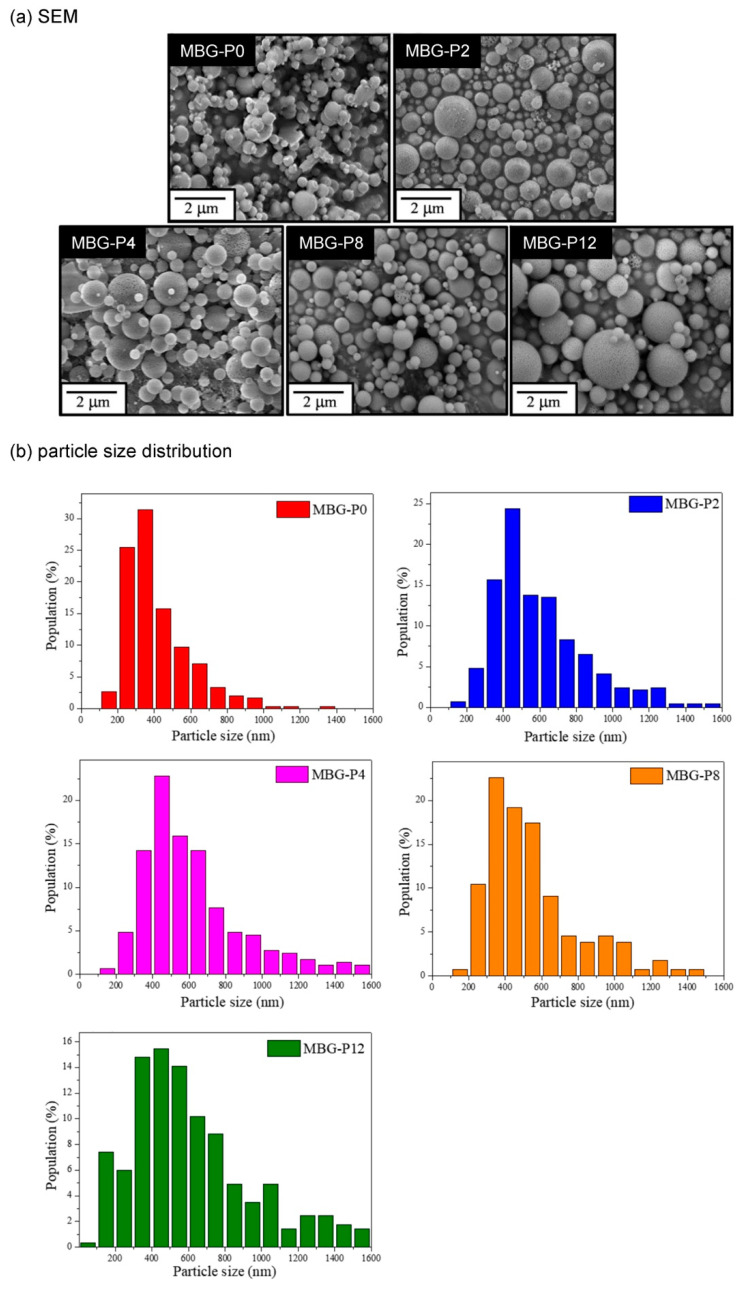
The FE- SEM images (**a**) and particle size distribution (**b**) of MBG adding different molecular weights of PEG.

**Figure 3:**
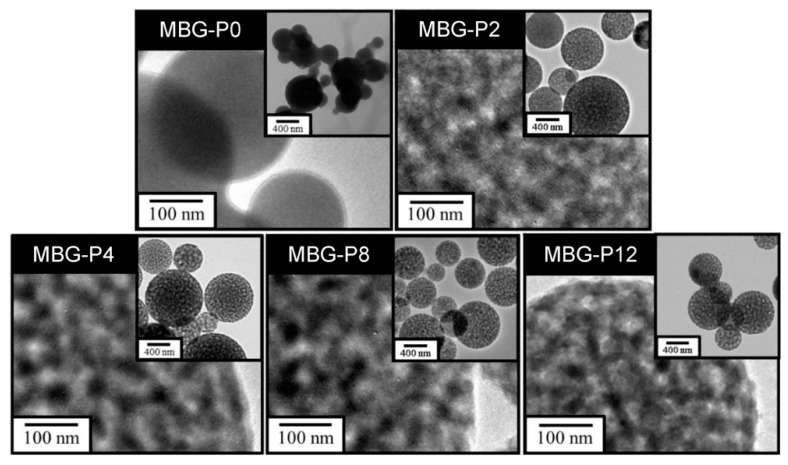
The FE-TEM images of MBG adding different molecular weights of PEG.

Figure 2a shows the SEM images of MBG prepared by adding different molecular weight of PFG. The surface of MBG-P0 presented to be smooth and was slightly agglomerated, but the particles of MBG-P2, MBG-P4, MBG-P8, and MBG-P12 were sphere-shaped with heterogenetic size, dispersed, and showed mesoporous structure, respectively. As shown in the Figure 2b, the average particle sizes of MBG particles were MBG-P0, 420 nm; MBG-P2, 620 nm; MBG-P4, 617 nm; MBG-P8, 677 nm; MBG-P12, 715 nm. Among them, the average particle size of MBG-P0 was the smallest, and the molecular weights of PEG added had a positive relationship with the average particle size of MBG particles. Figure 3 illustrates the TEM images, MBG-P0 (without surfactant PEG addition) was non-porous and without any pores; nevertheless, obvious and evenly distributed mesoporous structure can be observed in MBG-P2, MBG-P4, MBG-P8, and MBG-P12. The mesoporous properties were evaluated further by the BET method. The pore size increased from MBG-P2 to MBG-P12 from 3.136 nm to 3.149 nm; yet, it was only a slight change (Table 3). After adding PEG into the MBG particles, the specific surface area at least increased by 80%; in addition, the higher the molecular weight, the greater the specific surface area value. Note that, the specific surface area of MBG-P12 (121.53 m^2^/g) was 2.15 times that of MBG-P0 (56.48 m^2^/g) (Table 3).

**Figure 4:**
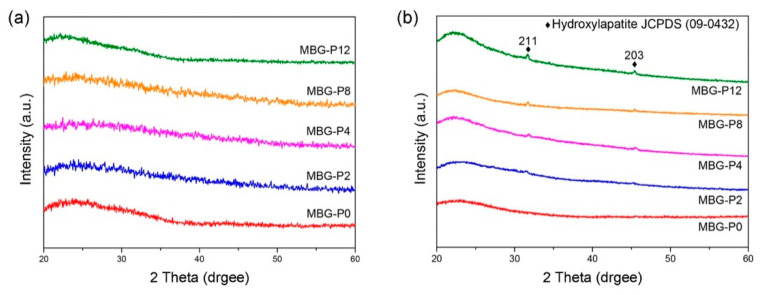
XRD patterns of MBG adding different molecular weights of PEG (**a**), and the bioactivity analysis results (XRD patterns) of MBG immersed in SBF for 24 h (**b**).

The result of XRD indicated that no prominent diffraction peak could be detected, and all MBG particles were amorphous (Figure 4a). The in vitro bioactivity analysis results are shown in Figure 4b. There were peaks at 31.8° and 45.3°, except for MBG-P0, which were consistent with the diffraction angle of hydroxyapatite (HA) in the JCPDS card number (No.09-0432). In addition, the peaks’ intensities increased slightly with the molecular weight of PEG. This shows a similar trend with the BET results (Table 3). According to the above BET and XRD results, it could be inferred that MBG-P12 exhibited the best in vitro bioactivity; thus, MBG-P12 will be investigated further in the following experiments.

### 3.2. Different Concentrations of Acetic Acid (CH_3_COOH)

**Figure 5:**
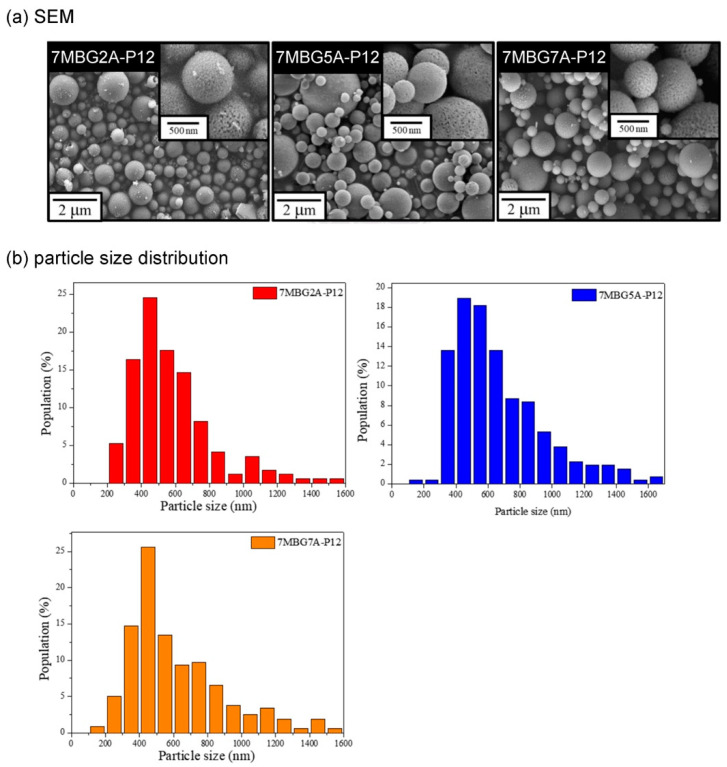
The FE- SEM images (**a**) and particle size distribution (**b**) of MBG adding different concentrations of CH_3_COOH.

**Figure 6:**
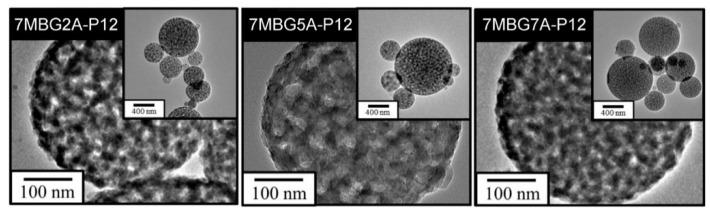
The FE-TEM images of MBG adding different concentrations of CH_3_COOH.

Figure 5a shows the SEM images. Similar to the previous part results, all MBG particles were spherical particles with different sizes. According to the particle analysis results (Figure 5b), the average particle sizes of 7MBG2A-P12, 7MBG5A-P12, and 7MBG7A-P12 were 613 nm, 684 nm, and 653 nm, respectively; yet, all of the obtained data were smaller than the 715 nm of MBG-P12. The TEM images of Figure 6 showed pronounced and evenly distributed mesoporous structure in 7MBG2A-P12, 7MBG5A-P12, and 7MBG7A-P12. The BET analysis results (Table 3) showed that the specific surface area and pore size exhibited no significant differences within 7MBG2A-P12, 7MBG5A-P12, and 7MBG7A-P12.

**Figure 7:**
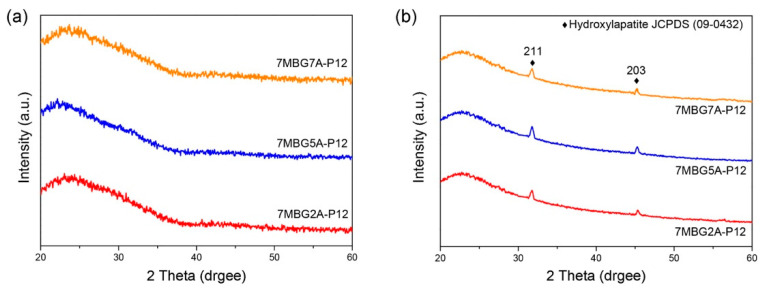
XRD patterns of MBG adding different concentrations of CH_3_COOH (**a**), and the bioactivity analysis results (XRD patterns) of MBG immersed in SBF for 24 h (**b**).

Figure 7a showed the corresponding XRD patterns where all the MBG particles were amorphous, regardless the concentrations of CH_3_COOH. The in vitro bioactivity analysis results are summarized in Figure 7b. 7MBG2A-P12, 7MBG5A-P12, 7MBG7A-P12 after immersion in SBF for 24 h had peaks at 31.8° and 45.3°, which were consistent with the diffraction angle of HA (JCPDS No.09-0432). Although there was no significant difference between MBG particles by changing the concentration of CH3COOH, 7MBG5A-P12 exhibited a slightly higher specific surface area and in vitro bioactivity (slightly larger peak intensity after SBF immersion); thus, 7MBG5A-P12 was investigated further by varying the concentration of PEG.

### 3.3. Different Concentrations of PEG

**Figure 8:**
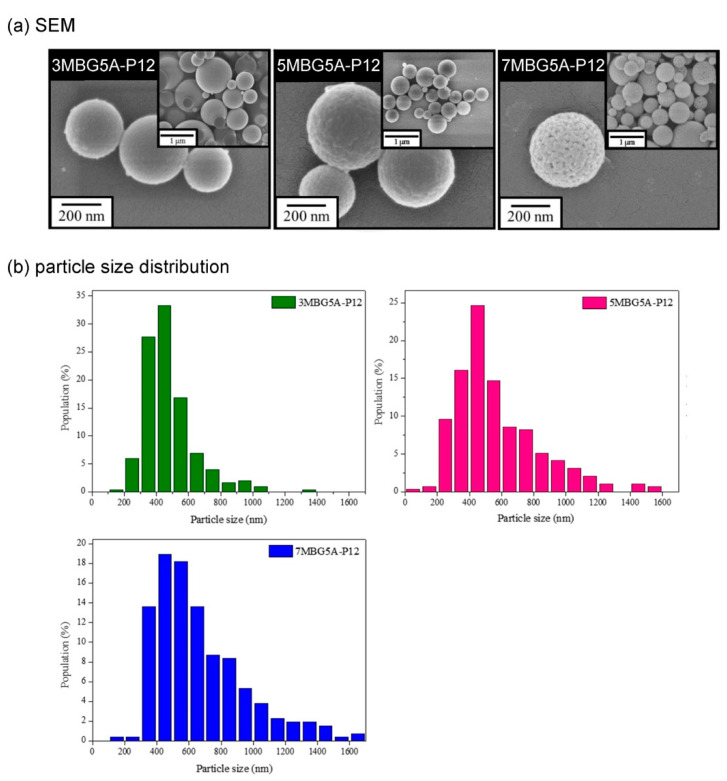
The FE- SEM images (**a**) and particle size distribution (**b**) of MBG adding different concentrations of PEG.

**Figure 9:**
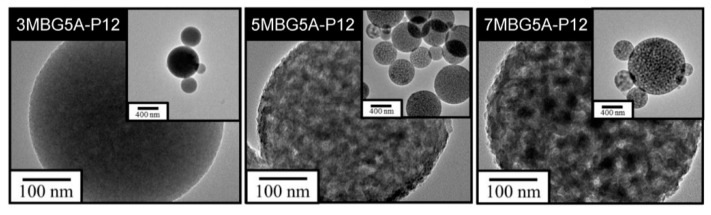
The FE-TEM images of MBG adding different concentrations of PEG.

Figure 8a showed the SEM images, all MBG particles were spherical and mesoporous. According to the particle analysis results (Figure 8b), the average particle sizes of 3MBG5A-P12, 5MBG5A-P12, and 7MBG5A-P12 were 484 nm, 570 nm, and 684 nm, respectively. The average particle sizes increased with increasing PEG concentrations. The TEM images are shown in Figure 9; it is interesting to note that 3MBG5A-P12 exhibited an obscure mesoporous structure, while 5MBG5A-P12 and 7MBG5A-P12 exhibited apparent mesoporous structure. It is suggested that the ambiguous mesoporous structure of 3MBG5A-P12 may be attributed to the relatively smaller pore size (1.924 nm) compared to those of 5MBG5A-P12 (3.481 nm) and 7MBG5A-P12 (3.152 nm). It should be pointed out that 3MBG5A-P12 exhibited the largest specific surface area (176.21 m^2^/g) investigated in the present study. The 5MBG5A-P12, however, possessed the largest pore size (3.481 nm) and the second highest specific surface area (173.93 m^2^/g, just slightly less than 176.21 m^2^/g). The BET analysis results are summarized in Table 3.

**Figure 10:**
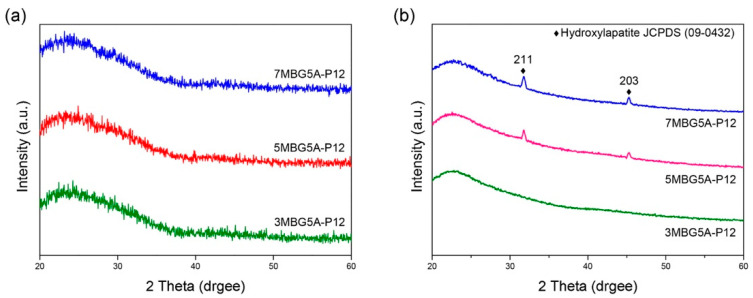
XRD patterns of MBG adding different concentrations of PEG (**a**), and the bioactivity analysis results (XRD patterns) of MBG immersed in SBF for 24 h (**b**).

**Figure 11:**
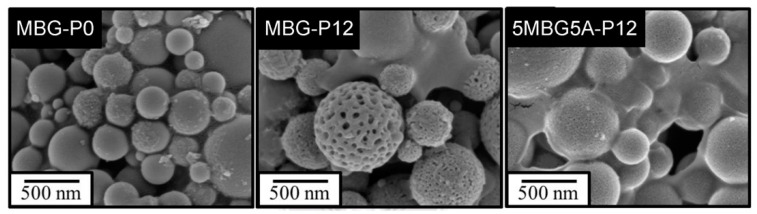
The FE-SEM images of MBG-P0, MBG-P12, and 7MBG5A-P12 immersed in SBF for 72 h.

Figure 10a indicates that MBG5A-P12 synthesized via adding different PEG concentrations were all amorphous. The in vitro bioactivity analysis results are shown in Figure 10b. 3MBG5A-P12, the one with the largest specific surface area, did not form HA. Whereas, 5MBG5A-P12 and 7MBG5A-P12 had peaks at 31.8° and 45.3°, which were probably identified as HA in the JCPDS card number (No.09-0432). In addition, 5MBG5A-P12 after immersion in SBF for 24 h exhibited the highest diffraction peaks compared to others. Figure 11 illustrated the SEM images of MBG-P0, MBG-P12, and 5MBG5A-P12 soaked in SBF for 72 h. In MBG-P0, very limited HA formation can be noted, while MBG-P12 and 5MBG5A-P12 had obvious formation of HA as thin lamellar structure. For MBG-P12, the mesoporous structure can still be observed, whereas for 5MBG5A-P12 it was covered uniformly by HA.

## 4. Discussion

Bioactive glass (BG) is an artificial biomaterial with high biocompatibility. Nowadays, Si–Ca–P ternary system, the most common BG component, has been widely used in biomedical-related fields [1,2]. Within the present study, in the synthesized mesoporous bioactive glass (MBG), Si was derived from the tetraethyl silicate (TEOS) in the precursor solution, Ca was from the calcium nitrate tetrahydrate (CN), and P was from triethyl phosphate (TEP) [8,30]. Shen et al. [30] added a polymer surfactant, F-127, to the precursor solution when synthesizing BG. The results indicated that BG synthesized without adding F-127 was spherical bioactive glass (SBG), but after adding F-127 was MBG. MBG had a relatively higher specific surface area and showed better bioactivity compared with SBG [30]. However, P123 and F127 were amphiphilic copolymers, not single components, so there would have been difficulties to study the detail for their physical properties [19,20]. Hence, the present study tried to add another polymer surfactant, polyethylene glycol (PEG), with good biocompatibility, to synthesize MBG. This experiment focuses on discussing the effect of PEG addition and evaluating whether there are differences in the mesoporous properties and in vitro bioactivity and mesoporous properties of MBG particles when different molecular weights and concentrations of PEG were added in MBG synthesis. Note that according to the preliminary experiments, the molecular weight of PEG with 16,000 or 20,000 exhibits excessive viscosity; thus, the molecular weight varying from 2000 to 12,000 was only considered in the present study. Additionally, literature pointed out that CH_3_COOH had better bioactivity than HCl when under the same pH [31]; moreover, CH_3_COOH can avoid cavitation of SP apparatus. Therefore, the present experiment changed the HCl as the hydrolysis catalyst to CH_3_COOH and evaluated the influence of different CH_3_COOH concentrations on the mesoporous structure of MBG particles.

The MBG particles synthesized under all PEG molecular weight conditions showed uneven spherical structures (Figure 2a), which was due to droplets collision during the atomization of the precursor solution [32]. There were noticeable and evenly distributed pores in MBG particles after adding PEG (Figure 3), but MBG-P0 (without added PEG) was a non-porous particle. The reasons were because SiO_2_ interacted with PEG, making PEG stay on the SiO_2_ surface and leading to in situ hydrolysis, forming pore-structures after pyrolysis [33]. The PEG can reduce surface tension; therefore, the surface tension of the precursor solution without added PEG was relatively large, so MBG-P0 formed by SP process was easily agglomerated. When PEG was added, the atomized droplets separated and formed spherical droplets, so the MBG particles (MBG-P2, MBG-P4, MBG-P8, MBG-P12) formed by pyrolysis exhibited good dispersibility and surface with pores. The addition of PEG alters the viscosity; therefore, the precursor solution’s viscosity increases when a specific PEG concentration was added, and the synthesized MBG also had larger particles [34,35] (Figure 2b). Additionally, the more comprehensive particle distribution range was due to the higher viscosity of the precursor solution, which led to a reduction in sheer force of the solution, so the particle size distribution finally became nonuniform [36]. PEG’s molecular weight affects the specific surface area of MBG particles because the pore structure formed by PEG was lost after in situ hydrolysis, so the specific surface area was altered only by the size of the external pores [33]. Therefore, MBG-P12 with the largest molecular weight exhibited the largest pore size, resulting in the largest specific surface area (Table 3).

When CH_3_COOH was substituted for HCl as the hydrolysis catalyst, the average particle size of MBG particles decreased (Figure 5b). This was because the surface tension of the solution with HCl was lower than that of CH_3_COOH [37]; therefore, the precursor solution’s viscosity was lower. However, increasing the concentration of CH_3_COOH reduced the surface tension of the solution [38], so the droplets were more extensive. As a result, the average particle size of 7MBG5A-P12 was larger than that of 7MBG2A-P12. Although CH_3_COOH helps increase the viscosity, when the viscosity exceeded the threshold, the viscosity instead decreased [39]. Owing to this report, the average particle size of 7MBG7A-P12 was smaller than 7MBG5A-P12. Nevertheless, the present study indicated that different CH_3_COOH concentrations had no significant difference in specific surface area and pore size, which means that the CH_3_COOH concentration did not affect the mesoporous-structure-generating performance of PEG (Table 3). Regarding the difference between HCl and CH_3_COOH on MBG synthesis, the average particle size of MBG-P12 (715 nm) was similar to 7MBG5A-P12 (684 nm). However, TEM images (Figure 6 and Figure 9) indicated that 7MBG5A-P12 had some particles with larger pore size and smaller average particle size (Table 3); precisely, because of this, the reaction area of 7MBG5A-P12 was relatively large, so the in vitro bioactivity also improved. Besides, the in vitro bioactivity analysis showed that 7MBG5A-P12 had a conspicuous peak (Figure 7b and Figure 10b), which proved that MBG synthesized using CH_3_COOH showed better in vitro bioactivity. In addition, 5MBG5A-P12 after immersion in SBF for 24 h exhibited the highest diffraction peaks compared to 3MBG5A-P12 and 7MBG5A-P12 (Figure 10b). Thus, it can be inferred that 5MBG5A-P12 was the MBG particles with the best mesoporous structure. The mesoporous structure of 5MBG5A-P12 cannot be revealed after immersion in SBF for 72 h. Individual MBG particles were interconnected by the formation of lamellar HA. Similar to the cold sintering process [40], densification of 5MBG5A-P12 can be noticed and is beneficial to the mechanical properties.

As shown by the FE-TEM images in Figure 3 and Figure 6, the spray-pyrolyzed particles with PEG additions were agglomerated with numerous bioactive glass nanocrystals that can serve as the nucleation sites for the formation of apatite. Though only two diffraction peaks were observed in the X-ray diffraction patterns, it was best identified as hydroxyapatite (JCPDS No.09-0432). The disappearance of other diffraction peaks was probably due to the amorphous background arising from the mesoporous bioactive glass. Similar behavior has been reported by Vlădescu et al. [41] who investigated the hydroxyapatite formation in SBF with the addition of silicon carbide (SiC). By XRD, only one characteristic peak corresponding to HA and SiC respectively can be observed due to the reduction of crystallinity. It should be also pointed out that the amorphous halo did not shift obviously to lower diffraction angle as the glass degraded and released ions in the immersion solution. As revealed by FE-SEM images in Figure 11, the dissolution of bioactive glass and precipitation of apatite only occurred on the surface of the MBG particles. The relatively small pore size (~2–3 nm shown in Table 3) constrained the fluid infiltration. Thus, the dissolution and degradation of MBG was limited and the microstructural integrity of MBG persisted.

Discussing the MBG particles synthesized through precursor solutions with different PEG concentrations (Figure 8a), the particle surfaces were smoother in low concentration and conversely were rougher in high concentration, presumably because PEG concentration led to the difference of pores formed after PEG pyrolysis. High PEG concentrations mean more PEG contained in the unit volume of the precursor solution so that the surface of the droplets created by the precursor solution contained more PEG. After pyrolysis, the PEG was removed and remained only apparent mesoporous, so the surface becomes rough. Besides, the higher the PEG concentrations, the larger the average particle size of the particles formed (Figure 8b), which was caused by the viscosity. When the PEG concentration is higher, the precursor solution viscosity increases [42], so the synthesized MBG particles become larger.

The SP processes can synthesize MBG in one step, and the MBG synthesized by adding PEG to the precursor was amorphous, spherical mesoporous material, and the pores were evenly distributed in the MBG. When the PEG molecular weights added in precursor solution were increased to 1200, the specific surface area of MBG improved resulting in MBG particles with better in vitro bioactivity. Replacing the hydrolysis catalyst of the precursor solution with CH_3_COOH also helped improve the biological activity; yet, PEG concentrations did not affect the in vitro bioactivity of MBG. Nonetheless, SiO_2_: CaO: P_2_O_5_ set in the present experiment was 80:15:5 mol% and the ratio of SiO_2_ is much higher than 60% of the commercialized bioactive glass “45S5”; therefore, the ratio of SiO_2_ needs to be adjusted in future works to obtain MBG with better in vitro bioactivity. Moreover, additive manufacturing (3D printing) or electrospinning can also be used to synthesize MBG scaffolds, allowing the cells to migrate, and showing tissue ingrowth, vascularization, and nutrient delivery in the future. Further investigations concerning the effect of pore size and longer immersion time may be required. In the present study, however, we have demonstrated that mesoporous bioactive glass particles with superior in vitro bioactivity can be synthesized successfully.

## 5. Conclusions

The spray pyrolysis (SP) process is an emerging process used in bioactive glass (BGs) synthesis. The SP process is a continuous one-pot processing, which can prepare low-cost and high purity mesoporous bioactive glass (MBG) at lower processing temperatures in a few hours. Besides, the synthesized MBG exhibits uniform composition and good mesoporous properties. Adding polyethylene glycol (PEG) to the precursor solution can successfully synthesize mesoporous bioactive glass (MBG). The MBG particles were spherical and mesoporous; besides, CH_3_COOH was a suitable hydrolysis catalyst for MBG synthesis. This study demonstrated that adding PEG can greatly increase the specific surface area of MBG, and the larger molecular weight of PEG was related to the higher specific surface area of MBG. A comprehensive consideration of the present study results prompt MBG synthesized under precursor solutions (1000 mL) by adding 5 g of PEG with 12,000 molecular weights, and 50 mL of CH_3_COOH (i.e., 5MBG5A-P12) exhibited the best in vitro bioactivity and mesoporous properties.

## Figures and Tables

**Table 1:** Medicament used in the present study.

Medicaments (Abbr.)	Manufacturer	Chemical Formula	Concentrations
Tetraethyl orthosilicate (TEOS)	Alfa Aesar Co., Massachusetts, MA, USA	Si(OC_2_H_5_)_4_	98.0 wt%
Calcium nitrate tetrahydrate (CN)	Showa Corporation, Tokyo, Japan	Ca(NO_3_)_2_·4H_2_O	98.5 wt%
Triethyl phosphate (TEP)	Alfa Aesar Co., Massachusetts, MA, USA	(C_2_H_5_)_3_PO_4_	>98.0 wt%
Hydrochloric acid	Acros Organics, New Jersey, NJ, USA	HCl	37.0 wt%
Polyethylene glycol (PEG)	Alfa Aesar Co., Massachusetts MA, USA	HO(CH_2_CH_2_O)_n_H	-
Acetic acid	Showa Corporation, Tokyo Japan	CH_3_COOH	99.7 wt%

**Table 2:** The synthesis conditions of mesoporous bioactive glass (MBG) and the sample code used in this study.

Acid		PEG		Sample Code
Medicaments	Concentrations	Molecular Weights	Concentrations	
I. Different Molecular Weights of PEG				
HCl	0.5 M	-	7 g/1000 mL	MBG-P0
HCl	0.5 M	2000	7 g/1000 mL	MBG-P2
HCl	0.5 M	4000	7 g/1000 mL	MBG-P4
HCl	0.5 M	8000	7 g/1000 mL	MBG-P8
HCl	0.5 M	12,000	7 g/1000 mL	MBG-P12
CH_3_COOH	25 mL/1000 mL	12,000	7 g/1000 mL	7MBG2A-P12
CH_3_COOH	50 mL/1000 mL	12,000	7 g/1000 mL	7MBG5A-P12
CH_3_COOH	75 mL/1000 mL	12,000	7 g/1000 mL	7MBG7A-P12
CH_3_COOH	50 mL/1000 mL	12,000	3 g/1000 mL	3MBG5A-P12
CH_3_COOH	50 mL/1000 mL	12,000	5 g/1000 mL	5MBG5A-P12
CH_3_COOH	50 mL/1000 mL	12,000	7 g/1000 mL	7MBG5A-P12

**Table 3:** The results of specific surface area and pore size of various MBG powders.

Sample Code	Specific Surface Area (m^2^/g)	Pore Size (nm)
MBG-P0	56.48	-
MBG-P2	102.08	3.136
MBG-P4	104.87	3.144
MBG-P8	108.24	3.146
MBG-P12	121.53	3.149
7MBG2A-P12	104.39	3.141
7MBG5A-P12	111.51	3.152
7MBG7A-P12	110.35	3.149
3MBG5A-P12	176.21	1.924
5MBG5A-P12	173.93	3.481
7MBG5A-P12	111.51	3.152
